# AI-Assisted Chemotherapy Regimen Selection and Its Effects on Clinical Outcomes and Adverse Drug Reactions: A Systematic Review

**DOI:** 10.7759/cureus.111337

**Published:** 2026-06-23

**Authors:** Abhishek Vadher, Swati Baraiya, Bobbadi Gajendra Siva Krishna Pavan Kumar, Utsav R Thakkar, Mohmadmahikhan F Pathan, Swathi Kambhatla, Faizkhan F Pathan, Pranay Gupta, Mayur Patel, Sujata Kambhatla

**Affiliations:** 1 Internal Medicine, Garden City Hospital, Garden City, USA; 2 Family Medicine, Bombay Hospital and Medical Research Centre, Mumbai, IND; 3 Internal Medicine, NRI Academy of Sciences, Guntur, IND; 4 Internal Medicine, Vedant Multispeciality Hospital, Ahmedabad, IND; 5 Internal Medicine, Byramjee Jeejeebhoy Medical College, Ahmedabad, IND; 6 Internal Medicine, Michigan State University, East Lansing, USA; 7 Infectious Disease, Moffitt Cancer Center, Tampa, USA; 8 Oncology, Garden City Hospital, Garden City, USA

**Keywords:** adverse reactions, ai, breast cancer, cancer, chemotherapy, lung cancer

## Abstract

Selection of optimal chemotherapy regimens remains a complex clinical challenge due to interpatient heterogeneity, evolving therapeutic options, and the limitations of population-based clinical guidelines. AI has emerged as a promising tool to support precision oncology by integrating multidimensional data to guide individualized treatment decisions. This systematic review evaluates the role of AI-based models in chemotherapy regimen selection, focusing on their impact on treatment efficacy and adverse drug reactions compared with conventional physician-driven decision-making.

A systematic literature search was conducted up to March 21, 2026. Studies evaluating AI-guided chemotherapy selection or treatment decision-support systems in cancer patients were included. The population, exposure, comparison, and outcomes (PECO) framework included cancer patients receiving AI-guided chemotherapy selection versus physician judgment or guideline-based care, with outcomes including survival, treatment response, and toxicity.

A total of 1,409 records were identified, with 15 studies meeting the inclusion criteria after screening and eligibility assessment. The included studies encompassed diverse malignancies, including breast, prostate, pancreatic, lung, head and neck, glioblastoma (GBM), hepatocellular carcinoma (HCC), nasopharyngeal carcinoma (NPC), and acute myeloid leukemia (AML). AI models utilized multimodal data sources, such as clinical variables, histopathology, imaging, and multi-omics datasets.

Across studies, AI-guided treatment selection was associated with improvements in several clinical outcomes, including overall survival, progression-free survival, and pathological response rates. Several models showed an enhanced ability to identify patients unlikely to benefit from specific chemotherapies, thereby enabling treatment de-escalation. Limited but notable evidence suggested reductions in treatment-related toxicity, particularly cardiotoxicity, when AI-guided strategies were employed. Most studies compared AI performance against physician clinical judgment or guideline-based approaches.

AI-assisted chemotherapy regimen selection shows considerable potential to improve treatment efficacy and personalize oncology care while reducing unnecessary toxicity. Although current evidence is largely retrospective and heterogeneous, findings consistently support AI as a valuable adjunct to clinical decision-making. Prospective validation and integration into real-world workflows are essential to establish its role in routine cancer care.

## Introduction and background

Cancer remains a leading cause of morbidity and mortality worldwide. The increasing complexity of cancer treatment, characterized by diverse patient prognoses and an expanding array of chemotherapeutic agents, requires advanced methods for optimizing therapeutic outcomes. Chemotherapy continues to be a core part of treatment for many cancers, including both solid tumors and hematological cancers. It is often used alongside targeted therapies, immunotherapy, or radiation to improve outcomes. However, choosing the right chemotherapy regimen is rarely simple. Clinicians must weigh tumor type and biology, patient fitness, organ function, existing medical conditions, and a constantly growing body of clinical trial evidence. These decisions carry significant consequences and cannot always be reduced to a single rule or guideline [1].

Organizations such as the National Comprehensive Cancer Network provide structured treatment recommendations, but these are built on population-level data. They cannot account for the full range of individual patient differences, nor can they easily incorporate the growing volume of molecular and clinical information generated in everyday cancer care. This gap between general guidelines and individual patient needs shows up clinically as poorly matched treatment choices, avoidable side effects, and outcomes that do not reflect the patient's actual potential for response [2].

Precision oncology developed as a response to this problem. Progress in genomics, molecular profiling, and imaging has greatly improved our ability to understand tumors at a biological level. The challenge now is not collecting data, it is making sense of it. Turning large amounts of complex patient information into clear, actionable treatment decisions remains one of the hardest problems in cancer care. Even experienced clinicians cannot reliably process dozens of interacting variables at once, consistently, across large patient populations [3]. This inherent complexity often leads to suboptimal treatment selection, variable efficacy, and significant patient toxicity.

AI approaches this differently. Using machine learning and statistical modeling, AI systems can detect patterns in large, complex datasets that humans would likely miss. In oncology, this has already been demonstrated across cancer detection, pathology analysis, imaging interpretation, and outcome prediction, and is now increasingly applied to treatment decision support [4,5]. One of the most promising uses is AI-guided chemotherapy selection. Models trained on combined datasets, including clinical records, pathology findings, genomic data, and treatment outcomes, can generate individualized predictions about which regimen is likely to work best for a given patient and which may cause unacceptable harm. This is especially useful when multiple guideline-approved options exist but carry different side-effect profiles. AI models that predict toxicity risk could also support dose planning, reducing serious side effects that necessitate dose reductions or treatment interruptions, outcomes that can, in turn, worsen prognosis [6]. This matters most in older patients or those with other illnesses, where the margin between effective and harmful treatment is narrow.

There is also potential in dynamic, ongoing decision-making. Cancer treatment in practice is not fixed; plans change as patients respond to treatment, tolerate it, or progress. Reinforcement learning models, designed to guide treatment decisions over time, could support dynamic adjustments based on a patient's evolving clinical condition rather than relying solely on information obtained at a single visit. That said, clinical use of AI in chemotherapy decision-making is still in its early stages. Most existing evidence comes from retrospective studies with wide variation in design, cancer type, and outcome measures, making it hard to draw firm conclusions. Questions about how models reach their decisions, whether they perform equally across different patient groups and health systems [7], and how to ensure data quality remain unresolved [8]. Without transparent, well-validated models, introducing AI into high-stakes clinical decisions could cause more harm than good. Reporting frameworks such as TRIPOD-AI, CONSORT-AI, and SPIRIT-AI, along with regulatory guidance, reflect growing agreement that AI in medicine must be scientifically sound and ethically responsible, while keeping clinicians meaningfully in control.

Studies have already suggested that AI has the potential to transform the healthcare system by helping physicians diagnose diseases, develop personalized treatment plans, and make complex decisions [9]. While individual studies have shown encouraging results, the field lacks a broad, critical overview of where AI-guided chemotherapy selection works and where evidence falls short. The primary objective of this systematic review is to comprehensively evaluate and synthesize published evidence on the application of AI-based approaches for chemotherapy regimen selection, treatment planning, and dosage adjustment across diverse cancer types. Specifically, this review aims to assess the effectiveness of AI systems in improving treatment outcomes, including response rates, progression-free survival, overall survival, and quality-of-life measures, while also examining their role in minimizing treatment-related toxicities and adverse events. Furthermore, the review seeks to evaluate the extent to which AI-driven tools facilitate personalized treatment strategies by integrating patient-specific clinical, pathological, genomic, and treatment-related data.

## Review

Methods

We conducted this systematic review in accordance with the Preferred Reporting Items for Systematic Reviews and Meta-Analyses (PRISMA) 2020 statement guidelines. 

Eligibility Criteria

The inclusion criteria for studies were as follows: (1) studies involving human participants with a confirmed diagnosis of cancer; (2) studies that evaluated the use of AI, ML, deep learning, reinforcement learning, or large language model (LLM)-based systems as decision-support tools for chemotherapy regimen selection, chemotherapy dose selection, treatment intensification, de-escalation, or optimization; (3) studies that included a comparator consisting of physician clinical judgment, multidisciplinary tumor board recommendations, standard clinical practice, or established treatment guidelines; and (4) studies that reported clinically relevant outcomes using statistical analyses, including but not limited to overall survival, PFS, disease-free survival (DFS), pathological complete response, treatment-related toxicity, treatment response, or other efficacy measures.

The exclusion criteria included in vitro studies using cancer cells and case studies. Systematic reviews and narrative reviews were excluded to avoid duplication of results.

Search Strategy

A comprehensive search of major databases was performed. We searched PubMed, Embase, Scopus, and the Cochrane Database. The search strategy employed Boolean operators with the following core terms: (“Artificial Intelligence” OR “Machine Learning” OR “Deep Learning”) AND (“Chemotherapy” OR “Antineoplastic Agents”) AND (“Cancer” OR “Neoplasm”). The detailed search strategy for each database is provided in Appendix 1.

Study Selection

All retrieved records were exported into the reference management software EndNote, and duplicates were removed prior to screening. A total of 1,409 records were identified (date of search: 03-21-2026). Records consisted of publications from the following databases: PubMed: 125, Embase: 856, Cochrane: 28, and Scopus: 400. Through EndNote software, 421 duplicates were removed, and 988 records were screened.

**Figure 1:**
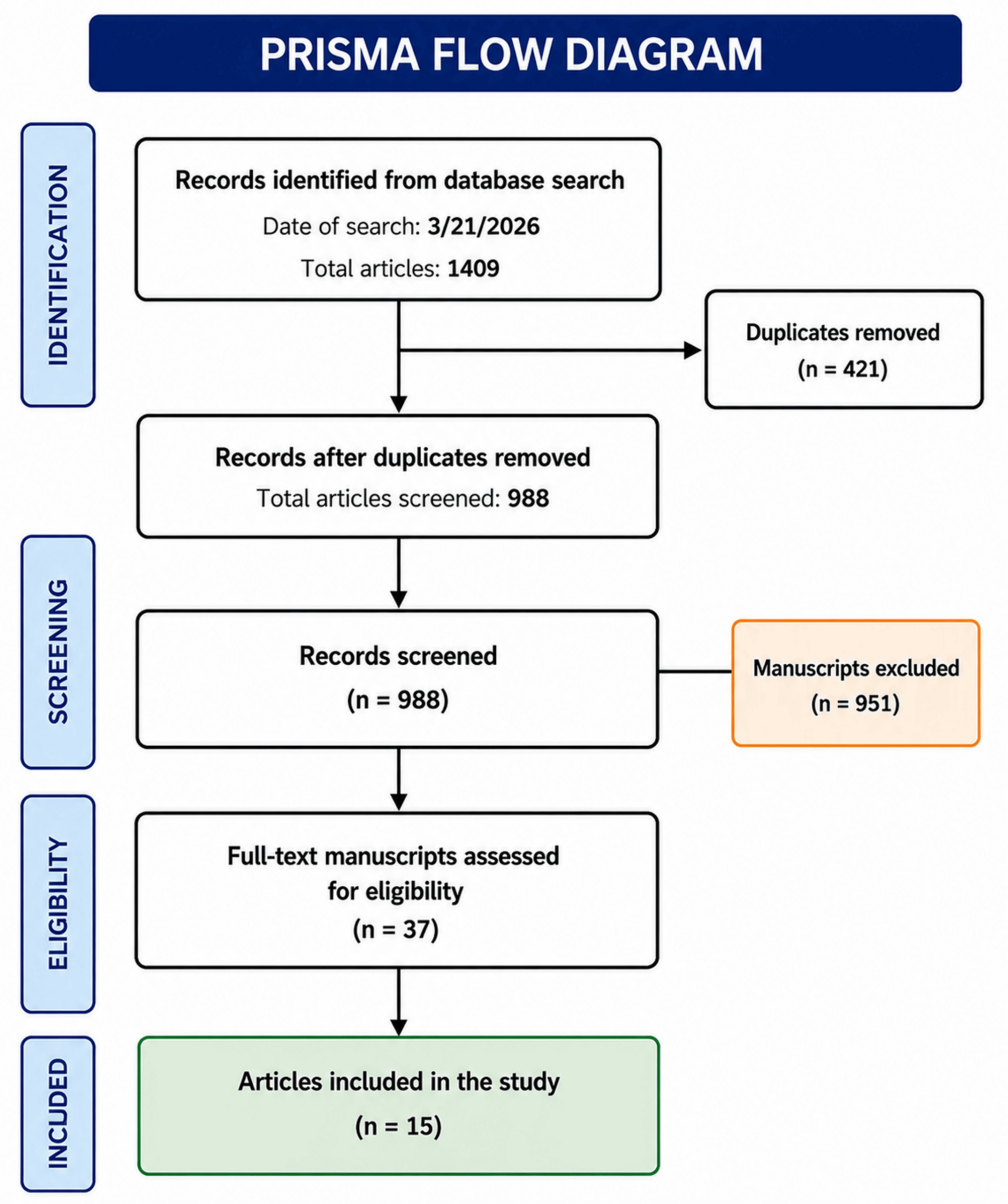
PRISMA flow diagram. PRISMA: Preferred Reporting Items for Systematic Reviews and Meta-Analyses.

Titles and abstracts were screened by two independent authors, Abhishek Vadher and Swati Baraiya, for eligibility. In case of discrepancy, a third author, Gajendra Pavan Kumar Bobbadi, helped make the decision about including versus excluding the article. A total of 37 articles underwent full-text review. Full manuscripts were reviewed by UT, FP, MP, AV, and SB for eligibility. Discrepancies were resolved by consensus. A total of 15 studies met the inclusion criteria and were included in the final qualitative synthesis (Figure 1).

Risk of Bias Assessment

Risk of bias of the included studies was assessed independently by two reviewers (AV and PG) using the Prediction Model Risk of Bias Assessment Tool + Artificial Intelligence (PROBAST+AI), an updated tool for evaluating prediction model studies using regression or AI methods [10]. PROBAST+AI assesses four key domains: participants, predictors, outcomes, and analysis. Each domain was rated as “low,” “high,” or “unclear” risk of bias [10]. Disagreements between reviewers were resolved by consensus with a third reviewer (GPKB). The risk of bias assessment is shown in Table 1.

**Table 1:** Risk-of-bias assessment using PROBAST+AI. PROBAST+AI: Prediction Model Risk of Bias Assessment Tool + Artificial Intelligence.

Study	Participants	Predictors	Outcome	Analysis	Overall
Medina S et al. (2026) [11]	Low	Low	Low	Unclear	Unclear
Zhu E et al. (2024) [12]	Low	Low	Low	High	High
Zhu E et al. (2024) [13]	Low	Low	Low	High	High
Hendifar AE et al. (2026) [14]	Low	Low	Low	Unclear	Unclear
Fraunhoffer N et al. (2024) [15]	Low	Low	Low	High	High
Howard FM et al. (2020) [16]	Low	Low	Low	High	High
Saad MB et al. (2025) [17]	Low	Low	Low	Unclear	Unclear
Tardini E et al. (2022) [18]	Low	Low	Low	High	High
Zhu E et al. (2024) [19]	Low	Low	Low	High	High
Yu K et al. (2017) [20]	Low	Low	Low	High	High
Yang X et al. (2024) [21]	Low	Low	Low	Unclear	Unclear
An C et al. (2025) [22]	Low	Low	Low	Unclear	Unclear
Liu Y et al. (2025) [23]	Low	Low	Low	High	High
Wang Y (2026) [24]	Unclear	Low	Low	High	High
Sanghvi A et al. (2025) [25]	High	Unclear	Unclear	High	High

Overall, 10 studies (66.7%) were classified as having a high risk of bias, and five studies (33.3%) were classified as having an unclear overall risk of bias. No study met all PROBAST+AI criteria for an overall low risk of bias. These findings highlight the methodological challenges that remain common in AI-based oncology prediction models, particularly with respect to model development, validation, and reporting standards.

Data Extraction

A standardized data extraction form was developed in Microsoft Excel and piloted on two randomly selected included studies before being applied to all studies. Data were extracted independently by two reviewers (AV and SB), and any discrepancies were resolved by consensus or by consultation with a third reviewer (GPKB). For each included study, the following data were extracted:

Study name: First author, year of publication, and study identifier.

AI model description: Type of AI model, such as BITES, DeepSurv, or CHAI; input data modalities, such as clinical variables, histopathology, or imaging; and the specific algorithm or framework used.

Comparison method: The comparator against which AI guidance was evaluated, such as physician clinical judgment or standard guidelines.

Patient population: Cancer type, stage, and sample size.

Study design: Retrospective cohort, case-control, prospective validation, or post-hoc analysis of randomized controlled trials.

Treatment efficacy outcomes: Reported measures such as OS, PFS, DFS, pathological complete response (pCR), HR, risk difference (RD), or restricted mean survival time (RMST).

Adverse drug reactions: Documented toxicity outcomes, such as cardiotoxicity, hematologic adverse events, or neuropathy, and how they were measured or compared between AI-guided and control groups.

Key findings: Main conclusions regarding the performance, benefit, or harm of AI-guided chemotherapy selection compared to the control.

Data Synthesis

Due to considerable variability in study designs, AI techniques, exposure and outcome measures, and reporting standards, a meta-analysis was deemed inappropriate; therefore, the results were synthesized narratively.

Results

Data from the 15 included studies are summarized in Table 2.

**Table 2:** Characteristics, study designs, and outcomes of the included studies. AC: Adjuvant chemotherapy; AML: Acute myeloid leukemia; APIC: Artificial intelligence pathology image classifier; BCSS: Breast cancer-specific survival; BCLC: Barcelona Clinic Liver Cancer; BITES: Balanced Individual Treatment Effect for Survival data; CHAI: Computational Histology Artificial Intelligence; ChIP-Seq: Chromatin immunoprecipitation sequencing; CRPC: Castration-resistant prostate cancer; CRT: Chemoradiotherapy; cTnI: Cardiac troponin I; DELICAITE: Deep Learning for Interaction and Covariate Analysis in Intra-arterial Therapy Selection; DFCI: Dana-Farber Cancer Institute; DFS: Disease-free survival; DQL: Deep Q-learning; DQN: Deep Q-network; DP: Dysphagia-related measure; dRMST: Difference in restricted mean survival time; ECE: Extracapsular extension; ER: Estrogen receptor; GBM: Glioblastoma; GEM: Gemcitabine; GvF: Gemcitabine versus FOLFIRINOX; HAIC: Hepatic arterial infusion chemotherapy; HER2: Human epidermal growth factor receptor 2; HNSCC: Head and neck squamous cell carcinoma; HR: Hazard ratio; ICI-Chemo: Immune checkpoint inhibitor plus chemotherapy; ICI-Mono: Immune checkpoint inhibitor monotherapy; IPTW: Inverse probability of treatment weighting; IQR: Interquartile range; LVEF: Left ventricular ejection fraction; MDA: Malondialdehyde; MDACC: MD Anderson Cancer Center; mFFX: Modified FOLFIRINOX; mHSPC: Metastatic hormone-sensitive prostate cancer; ML: Machine learning; mNSCLC: Metastatic non-small cell lung cancer; NCCN: National Comprehensive Cancer Network; N-MTLR: Neural network multitask logistic regression; NNT: Number needed to treat; NPC: Nasopharyngeal carcinoma; pCR: Pathological complete response; PDAC: Pancreatic ductal adenocarcinoma; PRES: Personalized Regimen Selection; PSA: Prostate-specific antigen; RAIRI: Response-adapted risk index; RD: Risk difference; R-GAT: Relation-aware graph attention network; RSF: Random survival forest; RT: Radiotherapy; SNB: Self-Normalizing Balanced; ST: Survival tree.

Study	AI model description	Comparison method	Patient population	Study design	Treatment efficacy outcomes	Adverse drug reactions	Key findings
Medina S et al. (2026) [11]	AI-based pathology image classifier. Digitized H&E-stained prostate core-needle biopsy specimens (imaging data); 286 patients from CHAARTED and 350 patients from NRG/RTOG 0521.	Standard clinical guidelines and physician clinical judgment.	Metastatic hormone-sensitive prostate cancer (mHSPC) and high-risk localized prostate cancer; maximum allowed PSA level of 150 ng/mL.	Retrospective analysis using data from two phase III randomized clinical trials (CHAARTED and NRG/RTOG 0521). Follow-up duration: median follow-up of 53.7 months for CHAARTED and 68.4 months for NRG/RTOG 0521.	Overall survival hazard ratio: OS HR 0.52 (95% CI 0.31-0.85); time to castration resistance: CRPC HR 0.48 (95% CI 0.33-0.71). Statistical significance: P=0.008 for OS and P<0.001 for CRPC.	Not reported.	APIC identifies patients who do not benefit from docetaxel, potentially reducing unnecessary chemotherapy toxicity.
Zhu E et al. (2024) [12]	Balanced Individual Treatment Effect for Survival data (BITES). Input data types: clinical variables, such as tumor size, lymph node status, and histological grade.	NCCN guidelines.	Sample size: 1,812 patients received chemotherapy, and 3,540 patients were in the control cohort.	Retrospective cohort study. Follow-up duration: median of 52 months (interquartile range: 30-80 months).	Treatment as per BITES: HR of 0.78 (95% CI 0.64-0.94), IPTW-adjusted HR of 0.74 (95% CI 0.59-0.93), RD of 12.40% (95% CI 8.01-16.90), and IPTW-adjusted RD of 11.50% (95% CI 7.16-15.80).	Not reported.	BITES improved treatment efficacy by reducing mortality by 12% and increasing survival time by 8 months.
Zhu E et al. (2024) [13]	Balanced Individual Treatment Effect for Survival data (BITES). Input data types used by the model: clinical variables, such as TNM stages and histological grades. Training dataset size and characteristics: 94,487 female breast cancer patients.	Physician-only decision-making.	Invasive breast cancer; stages determined by the 7th American Joint Committee on Cancer Staging Manual.	Retrospective cohort study. Follow-up duration: median 75 months (IQR 57-96 months).	IPTW-adjusted hazard ratio: 0.51 (95% CI 0.41-0.64); IPTW-adjusted risk difference: 21.46 (95% CI 18.90-24.01); IPTW-adjusted difference in restricted mean survival time: 21.51 months (95% CI 19.37-23.80). Statistical significance: OS (P<0.0001), BCSS (P<0.0001).	IPTW-adjusted HR for adverse effects: 0.47 (95% CI 0.17-0.78).	BITES led to a 21% reduction in mortality and extended survival by an additional 21 months over a 10-year period. BITES surpassed manual treatment decisions and other models in reducing mortality and improving survival rates.
Hendifar AE et al. (2026) [14]	Computational Histology AI (CHAI) platform. Input data types: whole-slide images of hematoxylin and eosin-stained diagnostic biopsies.	Not reported.	Advanced pancreatic ductal adenocarcinoma (PDAC). Sample size: development cohort=178; validation cohort=299.	Retrospective cohort study.	The GvF biomarker significantly predicted treatment outcomes, with interaction terms for time to next treatment or death (TNTD) (P=0.003) and OS (P=0.015).	Not reported.	The AI-based biomarker significantly improved TNTD and OS by selecting the most effective chemotherapy regimen.
Fraunhoffer N et al. (2024) [15]	Pancreas-View AI tool.	Standard clinical guidelines based on physician clinical judgment, focusing on general performance status and postoperative morbidities without considering molecular characteristics.	Pancreatic ductal adenocarcinoma (PDAC); stage: resected PDAC. Sample size: 178 patients in the mFFX group and 165 patients in the GEM group.	Retrospective cohort study.	Median DFS in the mFFX-sensitive group treated with mFFX was 50.0 months (stratified HR 0.31, 95% CI 0.21-0.44; P<0.001). In the GEM-sensitive group treated with GEM, median DFS was 33.7 months (stratified HR 0.40, 95% CI 0.17-0.59; P<0.001).	Not reported.	The Pancreas-View tool was effective in predicting drug response and improving survival outcomes when treatment matched AI-predicted sensitivity.
Howard FM et al. (2020) [16]	DeepSurv, RSF, and neural network multitask logistic regression (N-MTLR).	Standard clinical guidelines.	Resected head and neck squamous cell carcinoma (HNSCC) without positive margins or extracapsular extension (ECE). Sample size: 33,526 patients.	Retrospective cohort study.	DeepSurv (HR 0.76; 95% CI 0.69-0.84; P<0.001) and N-MTLR (HR 0.80; 95% CI 0.72-0.88; P<0.001).	Not reported.	AI models can effectively select patients for chemotherapy, improving treatment outcomes.
Saad MB et al. (2025) [17]	A-STEP (Attention-based Scoring for Treatment Effect Prediction).	Physician clinical judgment rather than standardized guidelines or tools.	Metastatic non-small cell lung cancer (mNSCLC), stage IV. Sample size: total of 2,300 patients across four cohorts: MDACC (n=750), Mayo (n=80), DFCI (n=1,077), and SU2C (n=393).	Retrospective cohort study.	Reduction in 3-month progression risk and improved 2-year progression-free survival (HR=0.60 for ICI-monotherapy; HR=0.58 for ICI-chemotherapy).	Not reported.	A-STEP improved weighted risk reduction by 13%-23% and showed improved 2-year progression-free survival.
Tardini E et al. (2022) [18]	Deep Q-learning (DQL). Training dataset size and characteristics: 402 patients for training and 134 patients for testing.	Physician treatment outcomes, which were aligned with state-of-the-art practices.	A total of 536 patients with oropharyngeal squamous cell carcinoma treated at MD Anderson Cancer Center between 2005 and 2013.	Retrospective cohort study.	OS improvement of +3.73% (95% CI -0.75% to +8.96%) and DP improvement of +0.75% (95% CI -4.48% to +6.72%).	Not reported.	The DQL model improved the survival rate by 3.73% compared with physician-only decisions. The DQL model provides a valid solution for dynamic treatment problems, improving upon physician-only decisions.
Zhu E et al. (2024) [19]	Balanced Individual Treatment Effect for Survival data (BITES).	Traditional T-learner and other machine learning-based methods.	Total sample size: 20,443 patients; 2,089 received RT and 18,354 received CRT. Patients were diagnosed with GBM as a primary cancer from 2005 to 2015 and received postoperative RT or CRT.	Retrospective cohort study.	Overall survival: HR 0.53 (95% CI 0.48-0.60); IPTW-adjusted HR 0.65 (95% CI 0.55-0.78); dRMST 7.92 months (95% CI 7.81-8.15). BITES had the best NNT (1.67, 95% CI 1.24-2.41), which was significantly better than DeepSurv (3.16, 95% CI 2.28-4.85) and ST (23.51, 95% CI 9.43-55.56).	Not reported.	The BITES model was effective in identifying patients who would benefit from CRT over RT, leading to better survival rates.
Yu K et al. (2017) [20]	Personalized Regimen Selection (PRES).	Original clinician-assigned regimens/best observed regimen group in the dataset.	Breast cancer. Sample size: 1,079 patients (A group: 139; TA group: 730; TxA group: 210).	Retrospective cohort study.	Pathological complete response (pCR): HER2-negative, 34.4% (95% CI 31.1%-39.5%); HER2/ER-negative, 49.2% (95% CI 44.3%-56.1%), significantly higher than the pCR of the original assignment.	Not reported.	The AI-based model, PRES, was estimated to have higher pCR rates than traditional treatments, especially for HER2-negative and ER-negative patients. This indicates improved treatment efficacy.
Yang X et al. (2024) [21]	Self-Normalizing Balanced (SNB) individual treatment.	National Comprehensive Cancer Network (NCCN) guidelines, representing standard clinical guidelines for chemotherapy recommendations in patients with triple-negative breast cancer (TNBC).	Triple-negative breast cancer (TNBC). Sample size: total of 10,070 patients; 1,568 in the non-chemotherapy group and 8,520 received chemotherapy.	Population-based retrospective cohort study.	IPTW-adjusted hazard ratio: 0.53 (95% CI 0.32-8.60); IPTW-adjusted risk difference: 12.90 (95% CI 6.99-19.01); IPTW-adjusted difference in restricted mean survival time: 5.54 months (95% CI 1.36-8.61). Statistical significance: P of log-rank test=0.0029; P of IPTW-adjusted log-rank test=0.0433 in the testing set; P of log-rank test=0.0490; P of IPTW-adjusted log-rank test=0.0284 in the external testing set.	Not reported.	The SNB model showed potential survival benefit compared with guideline-based approaches, with significant improvements in absolute risk reduction and restricted mean survival time. However, the wide confidence interval for the hazard ratio indicates uncertainty in the magnitude of effect.
An C et al. (2025) [22]	Deep Learning for Interaction and Covariate Analysis in Intra-arterial Therapy Selection (DELICAITE) model.	Physician clinical judgment.	Large hepatocellular carcinoma (HCC); BCLC stage C in 68% of cases. Total sample size: 900 patients; 453 in the TACE group and 447 in the HAIC group.	Retrospective cohort study.	AUCs of 0.756, 0.664, and 0.701 for the training, internal test, and external test sets, respectively; OS differences with P values <0.001.	The DELICAITE model can help reduce trial-and-error approaches and minimize the risk of adverse outcomes by providing evidence-based treatment recommendations.	The model demonstrated superior discriminative ability and accuracy in predicting progressive disease, and patients classified by the model showed significantly longer overall survival.
Liu Y et al. (2025) [23]	RAIRI, a dynamic and multidimensional model.	Standard clinical guidelines.	Nonmetastatic nasopharyngeal carcinoma (NPC). Total sample size: 2,148 patients.	Retrospective cohort study.	For RAIRI-low-risk patients: HR 1.10 (95% CI 0.37-3.28; P=0.860). For RAIRI-high-risk patients: HR 0.40 (95% CI 0.22-0.73; P=0.003).	The study implies that RAIRI could reduce adverse drug reactions by identifying low-risk patients who do not benefit from AC, thus avoiding unnecessary chemotherapy.	RAIRI demonstrated high prognostic accuracy and outperformed conventional models, suggesting improved treatment efficacy by better selecting patients for AC.
Wang Y (2026) [24]	Deep learning, including graph neural networks and deep reinforcement learning. Specific algorithms/frameworks: relation-aware graph attention network (R-GAT) and deep Q-networks (DQN). Input data types: multi-omics data, including genome-wide methylation profiles, transcriptome data, and histone modification ChIP-Seq profiles; and clinical variables, including patient baseline features, tumor characteristics, cardiac function indicators, and oxidative stress markers.	Standard NCCN guideline regimens, representing the current standard of care.	Breast cancer and lymphoma. Sample size: 327 patients.	Retrospective cohort study. AI strategy was compared against standard regimens retrospectively.	Tumor response rate showed a 2% improvement in the AI model (P=0.634).	Cardiac toxicity event rate was reduced from 28.3% to 14.7% (P<0.001). Cardiac toxicity events were defined as LVEF decline >10% or cTnI >0.5 ng/mL. LVEF decline magnitude was reduced by 50.6% (P<0.001), peak cTnI was reduced by 54.8% (P<0.001), and MDA level was reduced by 32.8% (P<0.001).	The AI strategy reduced cardiac toxicity events by 48.1% while maintaining a comparable tumor response rate.
Sanghvi A et al. (2025) [25]	OpenEvidence, ChatGPT-4 (OpenAI), Gemini 2.5 (Google), and Claude Opus (Anthropic).	Physician clinical judgment and experience.	Acute myeloid leukemia (AML). Sample size: 20 patients.	Retrospective chart review.	80% concordance with actual clinical management by physicians. Overall prognosis: 80% accuracy compared with physicians.	Not reported.	Comparable clinical management and prognosis, but low accuracy in non-clinical factors.

Study Characteristics and Comparators

This review included 15 studies evaluating AI as a guiding tool for chemotherapy regimen selection, escalation/de-escalation, or treatment intensification decisions across prostate cancer, breast cancer, pancreatic ductal adenocarcinoma (PDAC), head and neck cancers, metastatic non-small cell lung cancer (mNSCLC), glioblastoma (GBM), hepatocellular carcinoma (HCC), nasopharyngeal carcinoma (NPC), and acute myeloid leukemia (AML). Study designs were predominantly retrospective, including registry-based cohorts, retrospective analyses of randomized controlled trial biospecimens, and retrospective multi-cohort validations. Input modalities included digitized H&E/whole-slide pathology, transcriptomic signatures, routine clinical variables, radiomic/clinical features for reinforcement learning, multi-omics plus clinical phenotypes, and structured prompts to LLMs. Most studies compared AI-guided recommendations against standard clinical practice, including physician judgment and/or guideline-based care, or against observed outcomes under real-world treatments, frequently operationalized as “treatment consistent with model recommendation” versus “inconsistent.” A minority compared AI-guided approaches against alternative modeling baselines, such as T-learner-style comparators or other machine learning survival models. The heterogeneity in comparators and decision tasks, including regimen selection, adding chemotherapy, and dynamic treatment sequences, limits direct cross-study comparability.

Findings by Cancer Type

Prostate cancer: Medina S et al. evaluated an AI pathology image classifier (APIC) using digitized H&E-stained biopsy specimens from two phase III trial cohorts (CHAARTED, n=286; NRG/RTOG 0521, n=350) [11]. In metastatic hormone-sensitive prostate cancer (mHSPC), APIC identified a docetaxel-benefit subgroup with an OS HR of 0.52 (95% CI 0.31-0.85; P=0.008) and time to castration resistance HR of 0.48 (95% CI 0.33-0.71; P<0.001), supporting a potential de-escalation use case for non-benefiters. Breast cancer: Zhu E et al. reported BITES-based guidance using clinical variables in large retrospective cohorts, with an HR of 0.78 (95% CI 0.64-0.94), IPTW-adjusted HR of 0.74 (95% CI 0.59-0.93), RD of 12.40% (95% CI 8.01-16.90), and IPTW-adjusted RD of 11.50% (95% CI 7.16-15.80), with a median follow-up of 52 months [12]. Zhu E et al. additionally reported, in 94,487 patients, an IPTW-adjusted HR of 0.51 (95% CI 0.41-0.64), IPTW-adjusted RD of 21.46 (95% CI 18.90-24.01), and IPTW-adjusted dRMST of 21.51 months (95% CI 19.37-23.80), with OS/BCSS P<0.0001; an adverse effects HR of 0.47 (95% CI 0.17-0.78) was also reported [13]. Yu K et al. (PRES) used gene-expression profiles to guide selection among neoadjuvant chemotherapy regimens and reported estimated pathological complete response (pCR) improvements versus clinician-assigned/best observed regimens: HER2-negative pCR of 34.4% (95% CI 31.1-39.5) and HER2/ER-negative pCR of 49.2% (95% CI 44.3-56.1) [20]. Yang et al. (SNB model), in patients with triple-negative breast cancer (TNBC; n=10,070), reported an IPTW-adjusted HR of 0.53 (95% CI 0.32-8.60), indicating a favorable but imprecise estimate. The model also demonstrated significant improvements in IPTW-adjusted RD of 12.90% (95% CI 6.99-19.01) and IPTW-adjusted dRMST of 5.54 months (95% CI 1.36-8.61), with statistically significant log-rank tests in the internal and external testing sets [21].

Toxicity optimization and LLM decision support: Wang Y et al. reported a deep learning strategy using graph neural networks and deep reinforcement learning that was associated with a reduction in cardiac toxicity events from 28.3% to 14.7% (P<0.001), while maintaining comparable tumor response, with a 2% improvement (P=0.634). The magnitude of left ventricular ejection fraction (LVEF) decline was reduced by 50.6% [24]. Pancreatic cancer: Hendifar AE et al. (CHAI platform; whole-slide images) reported a significant treatment-outcome interaction for the GvF biomarker with time to next treatment or death (P=0.003) and overall survival (P=0.015) in advanced PDAC, implying a predictive biomarker for choosing the more effective regimen [14]. Fraunhoffer N et al. (Pancreas-View transcriptomic tool) reported that matched regimen administration improved DFS: in the mFFX-sensitive group treated with mFFX, median DFS was 50.0 months, with a stratified HR of 0.31 (95% CI 0.21-0.44; P<0.001); in the GEM-sensitive group treated with GEM, median DFS was 33.7 months, with a stratified HR of 0.40 (95% CI 0.17-0.59; P<0.001) [15].

Head and neck cancers: Howard FM et al. evaluated machine learning survival models, including DeepSurv, RSF, and N-MTLR, to guide adjuvant chemotherapy selection in resected head and neck squamous cell carcinoma (n=33,526) and reported DeepSurv HR of 0.76 (95% CI 0.69-0.84; P<0.001) and N-MTLR HR of 0.80 (95% CI 0.72-0.88; P<0.001) [16]. Tardini E et al. applied deep Q-learning to sequential therapy decisions in oropharyngeal squamous cell carcinoma (n=536; training set, n=402; test set, n=134), reporting an OS improvement of +3.73% (95% CI −0.75% to +8.96%) and dysphagia-related measure improvement of +0.75% (95% CI −4.48% to +6.72%) versus physician decisions [18].

Metastatic non-small cell lung cancer: Saad MB et al. (A-STEP; multiple cohorts totaling n=2,300) reported improved 2-year PFS, with an HR of 0.60 for ICI-monotherapy and an HR of 0.58 for ICI-chemotherapy, alongside reduced 3-month progression risk and weighted risk reduction improvements [17]. Liu Y et al. (RAIRI; n=2,148) reported that adjuvant chemotherapy benefit differed by RAIRI risk: RAIRI-low-risk HR of 1.10 (95% CI 0.37-3.28; P=0.860) versus RAIRI-high-risk HR of 0.40 (95% CI 0.22-0.73; P=0.003) [23].

Glioblastoma: Zhu E et al. (n=20,443; RT, n=2,089; CRT, n=18,354) reported an overall survival HR of 0.53 (95% CI 0.48-0.60), IPTW-adjusted HR of 0.65 (95% CI 0.55-0.78), dRMST of 7.92 (95% CI 7.81-8.15), and NNT of 1.67 (95% CI 1.24-2.41), with BITES outperforming DeepSurv and survival-tree comparators on NNT and showing significant Kaplan-Meier separation in “consistent versus inconsistent” groups [19].

Hepatocellular carcinoma: An C et al. (DELICAITE; n=900) reported AUC values of 0.756 in the training set, 0.664 in the internal test set, and 0.701 in the external test set, as well as significant OS differences (P<0.001) between model-stratified groups [22].

Acute myeloid leukemia: Sanghvi A et al. reported an AML chart-review evaluation of multiple AI tools/LLMs with approximately 80% concordance with physician management and approximately 80% accuracy for prognosis, with limitations in non-clinical factors [25].

Heterogeneity and Narrative Synthesis

There was substantial heterogeneity in cancer types and treatment pathways; AI objectives, including regimen selection, escalation, and sequential decision optimization; input modalities, including pathology, transcriptomics, clinical variables, multi-omics, and LLM prompts; comparators; and endpoints, including pCR, OS, DFS, PFS, TNTD, time to CRPC, and AUC. Given this clinical and methodological diversity and the predominance of observational designs, pooling effect sizes would be misleading; therefore, a qualitative synthesis is more defensible in this manuscript.

Discussion

AI-Guided Chemotherapy Selection as a Positive Force in Cancer Care

The included studies [11-25] collectively support a clear, optimistic message: AI is increasingly functioning as an enabling technology for precision oncology, particularly where clinicians must balance competing aims, maximizing tumor control while minimizing avoidable toxicity [26]. The included studies span multiple cancers and AI modalities, including computational pathology, transcriptomics, causal inference/survival learning, reinforcement learning, and early LLM decision support, illustrating that AI’s value is not tied to a single algorithm family but to its shared capability of integrating high-dimensional information to individualize treatment choices.

To translate this promise into durable patient benefit, the broader oncology and clinical AI communities increasingly emphasize rigorous clinical evaluation and transparent reporting. The CONSORT-AI and SPIRIT-AI extensions provide AI-specific requirements for trial reports and protocols, respectively, which are crucial for moving beyond retrospective performance and toward prospective impact evaluation [27,28]. The DECIDE-AI guideline further highlights the importance of early-stage, real-world clinical evaluation focusing on safety, human factors, and feasibility before large-scale trials [29].

How AI Enhances Chemotherapy Decisions Beyond Guidelines

A central clinical advantage of AI decision support in chemotherapy selection is its ability to model heterogeneity of treatment effect, identifying which subgroups are most likely to benefit from intensification, such as adding chemotherapy or choosing a more aggressive regimen, and which may safely avoid it. Within the included studies, this is reflected in multiple patterns: predictive pathology-derived biomarkers for benefit, such as docetaxel benefit stratification; regimen sensitivity tools in pancreatic cancer; and individualized treatment-effect modeling that can support escalation/de-escalation decisions across diverse disease contexts. These applications align with the broader view that AI’s most meaningful contributions in oncology arise when it helps clinicians move from population-average recommendations to patient-specific benefit-harm trade-offs [30].

A major reason this is feasible is that AI can extract predictive signals from data that are either already routine, such as H&E histology and standard clinical variables, or becoming more widely available, such as multi-omics, thereby lowering the barrier to precision treatment selection. A widely cited example of this “routine data to actionable biomarker” pathway is histology-based deep learning to infer molecular features relevant to treatment selection. For instance, deep learning has been shown to predict microsatellite instability from H&E histology in gastrointestinal cancer [31], potentially expanding access to therapy-relevant information when molecular testing is incomplete or delayed [32].

In practice, such AI-driven stratification can support several positive clinical outcomes that the included evidence base consistently points toward: (1) better matching of regimen intensity to patient benefit; (2) reduced exposure to ineffective chemotherapy, and therefore reduced avoidable toxicity [33]; and (3) more efficient therapeutic decision-making in data-rich settings where purely manual synthesis is increasingly difficult.

Positive Impact Pathways: Efficacy, Toxicity Reduction, and Dynamic Adaptation

An important strength of AI-guided chemotherapy selection is that it does not need to be framed as “AI replacing clinicians.” A better and more clinically acceptable framing is augmentation: AI supports oncologists by providing probabilistic benefit estimates, identifying high-risk/high-benefit subgroups, and surfacing patterns that may not be apparent in routine assessment. This human-AI partnership framing is consistent with broader synthesis work describing “high-performance medicine” as the convergence of human judgment and AI-enabled analytic capacity, which is particularly valuable in complex decision environments such as oncology [32].

Included studies suggest several distinct positive use cases such as:

Regimen selection and matching: Models that classify likely regimen sensitivity, such as in PDAC, illustrate AI’s ability to support the “right regimen for the right patient,” potentially improving disease control while limiting unnecessary toxicity from ineffective options.

Chemo escalation/de-escalation: Decision support that identifies likely non-benefiters to intensification, such as adding docetaxel or adding chemotherapy to another modality, offers a patient-centered benefit by avoiding toxicity and treatment burden when incremental benefit is unlikely.

Toxicity-aware optimization: The cardio-oncology-oriented included study emphasizes a particularly compelling positive vision: AI systems that maintain oncologic efficacy while meaningfully reducing serious adverse events. Even when early and retrospective, this direction matters because chemotherapy decision-making is intrinsically based on benefit-harm balancing, and AI is well suited to formalize and individualize that balance.

Dynamic treatment strategies: Reinforcement learning-oriented approaches point toward adaptive, longitudinal decision support, where therapy selection is iteratively updated using evolving patient states. This is clinically aligned with how oncology care unfolds, especially in metastatic disease.

The Emerging Role of Multimodal and LLM-Based Systems

While many oncology AI systems have historically focused on single-modality prediction tasks, the field is rapidly moving toward multimodal and tool-augmented systems that can handle the real-world complexity of oncology decision-making, including clinical text, pathology, imaging, genomics, and guidelines. A recent open-access study in Nature Cancer described the development and validation of an autonomous AI agent built around a LLM coordinated with specialized precision-oncology tools and retrieval, explicitly aiming to support personalized clinical decision-making in realistic multimodal patient scenarios [34].

At the same time, oncology-specific guidance urges careful, evidence-based integration of language models into clinical practice to preserve safety, reliability, and trust, while still recognizing their potential benefits in navigating unstructured clinical text and guideline-heavy workflows [35]. This is directly relevant to the included LLM-focused study [25], which illustrates early feasibility signals, such as concordance, but also highlights that LLM use in oncology decision support must be grounded, evaluated, and implemented with strong safeguards.

A practical way to keep this discussion optimistic is to emphasize that LLMs are best positioned as workflow accelerators and integration layers, while “clinical-grade” recommendations should be anchored to validated tools, curated knowledge sources, and guideline-concordant retrieval rather than free-form generation. This view is also consistent with broader oncology perspectives on AI’s role in therapeutic decision-making, highlighting opportunities in personalization while underscoring the need for clinical validation [36].

From Algorithm Development to Clinical Adoption

A positive future for AI-guided chemotherapy selection depends not only on model ingenuity but also on reproducibility, interpretability, and transferable evaluation. Reporting frameworks are therefore not merely compliance tools; they are enablers of clinical adoption.

For prediction-model reporting, TRIPOD+AI provides harmonized guidance for transparent reporting of prediction model studies using regression or machine learning and explicitly supersedes TRIPOD 2015 for AI-era models [37]. For structured critical appraisal, PROBAST supports systematic assessment of risk of bias and applicability in prediction model studies [38]. Recognizing that AI introduces additional design and evaluation pitfalls, PROBAST+AI updates this framework for prediction models using regression or AI methods and is intended to improve quality assessment across stakeholders, including developers, reviewers, and clinicians [39].

Regulatory and governance guidance should be framed as accelerators of beneficial AI, not barriers. The U.S. Food and Drug Administration highlights that AI/ML technologies can transform health care but also require attention to the complexity and iterative nature of ML product development; its Good Machine Learning Practice (GMLP) guidance emphasizes safe, effective, high-quality AI/ML medical devices and total product lifecycle thinking [37]. The International Medical Device Regulators Forum SaMD Clinical Evaluation framework further clarifies expectations for clinical evaluation of software as a medical device, which is highly relevant when AI tools are intended to guide treatment selection [40,41].

Finally, the WHO ethics and governance guidance underscores that AI holds promise for improving diagnosis, treatment, research, and drug development, but should embed ethics and human rights into design, deployment, and use [42]. This guidance supports a positive framing: AI can expand access to individualized cancer care if governance ensures accountability, transparency, and protection of patient interests.

Even in a discussion focused on AI’s positive role, it strengthens credibility to acknowledge that the dominant evidence base in the included studies is retrospective and highly heterogeneous in disease sites, endpoints, and comparators. This does not negate promise; rather, it clarifies the next milestone: prospective evaluation of clinical impact, ideally with multicenter validation and clear benefit-harm endpoints.

## Conclusions

AI-assisted chemotherapy regimen selection shows significant potential to improve treatment efficacy and personalize oncology care while reducing unnecessary toxicity. Although current evidence is largely retrospective and heterogeneous, findings consistently support AI as a valuable adjunct to clinical decision-making. Further prospective studies with large patient populations across different cancer types are needed to establish its role in routine cancer care.

## Appendices

Appendix 1

AI (Artificial intelligence); AML (Acute myeloid leukemia); APIC (Artificial intelligence pathology image classifier); AUC (Area under the curve); BCSS (Breast cancer-specific survival); BITES (Balanced Individual Treatment Effect for Survival data); CI (Confidence interval); CRPC (Castration-resistant prostate cancer); CRT (Chemoradiotherapy); DFS (Disease-free survival); dRMST (Difference in restricted mean survival time); GBM (Glioblastoma); GEM (Gemcitabine); H&E (Hematoxylin and eosin); HAIC (Hepatic arterial infusion chemotherapy); HCC (Hepatocellular carcinoma); HER2 (Human epidermal growth factor receptor 2); HR (Hazard ratio); ICI (Immune checkpoint inhibitor); IPTW (Inverse probability of treatment weighting); LLM (Large language model); LVEF (Left ventricular ejection fraction); mFFX (Modified FOLFIRINOX); mHSPC (Metastatic hormone-sensitive prostate cancer); mNSCLC (Metastatic non-small cell lung cancer); N-MTLR (Neural network multitask logistic regression); NCCN (National Comprehensive Cancer Network); NNT (Number needed to treat); NSCLC (Non-small cell lung cancer); NST (Neoadjuvant systemic therapy); NPC (Nasopharyngeal carcinoma); OS (Overall survival); pCR (Pathological complete response); PDAC (Pancreatic ductal adenocarcinoma); PFS (Progression-free survival); PRES (Personalized Regimen Selection); RAIRI (Response-adapted risk index); RD (Risk difference); RSF (Random survival forest); RT (Radiotherapy); SNB (Self-Normalizing Balanced); TACE (Transarterial chemoembolization); TNBC (Triple-negative breast cancer); TNTD (Time to next treatment or death).
